# Synergy of Urban Heat, Pollution, and Social Vulnerability in One of America's Most Rapidly Growing Cities: Houston, We Have a Problem

**DOI:** 10.1029/2024GH001079

**Published:** 2024-09-04

**Authors:** Andrew Blackford, Trent Cowan, Udaysankar Nair, Christopher Phillips, Aaron Kaulfus, Brian Freitag

**Affiliations:** ^1^ Department of Atmospheric and Earth Science The University of Alabama in Huntsville Huntsville AL USA; ^2^ Earth System Science Center The University of Alabama in Huntsville Huntsville AL USA; ^3^ National Aeronautical and Space Administration Marshall Space Flight Center Huntsville AL USA

**Keywords:** Houston, pollution, heat stress, urban heat island, urban pollution island, social vulnerability

## Abstract

During the first two decades of the twenty‐first century, we analyze the expansion of urban land cover, urban heat island (UHI), and urban pollution island (UPI) in the Houston Metropolitan Area (HMA) using land cover classifications derived from Landsat and land/aerosol products from NASA’s Moderate Resolution Imaging Spectroradiometer. Our approach involves both direct utilization and fusion with in situ observations for a comprehensive characterization. We also examined how social vulnerability within the HMA changed during the study period and whether the synergy of UHI, UPI, and social vulnerability enhances environmental inequalities. We found that urban land cover within the HMA increased by 1,345.09 km^2^ and is accompanied by a 171.92 (73.93) % expansion of the daytime (nighttime) UHI. While the UPI experienced an overall reduction in particulate pollution, the magnitude of change is smaller compared to the surroundings. Further, the UPI showed localized enhancement in particulate pollution caused by increases in vehicular traffic. Our analysis found that the social vulnerability of the HMA urban regions increased during the study period. Overall, we found that the urban growth during the first two decades of the twenty‐first century resulted in a synergy of UHI, UPI, and social vulnerability, causing an increase in environmental inequalities within the HMA.

## Key Points

- The rapid urbanization of Houston expands the urban heat and pollution islands, raising exposure to high temperatures and poor air quality
- Communities with highest social vulnerability are most impacted by the adverse effects of urban heat and pollution islands
- The Clean Air Act’s efficacy is locally diminished by Houston’s urban pollution island

## 1 Introduction

The Houston Metropolitan Area (HMA), Texas, one of the top 10 most populous metropolitan areas in the United States (U.S.), is ethnically and racially diverse but also faces severe racial and economic segregation (Emerson et al., 2012). Approximately 24% and 37% of HMA's high‐income and low‐income households, respectively, are located within corresponding neighborhoods linked to historic redlining practices (Lane et al., 2022; Nicholson, 2017). Such segregation contributes to environmental inequities, including heightened heat stress, pollution, flood risk, and reduced green space access (Bullard, 1983; Fry & Taylor, 2012; Logan et al., 2021; Waren, 2013). Prior work (Ulpiani, 2021) has found that urban regions have increased air temperature and pollution compared to rural regions, termed Urban Heat Islands (UHI) and Urban Pollution islands (UPI) respectively. Additionally, social vulnerability refers to socioeconomic factors that increase the risk of adverse health effects from environmental conditions (Mah et al., 2023). Understanding synergies between the UHI, UPI, and social vulnerability are therefore important for equitable and healthy urban growth (H. Li et al., 2018; Logan et al., 2021; Streutker, 2003; Ulpiani, 2021). Additionally, urban growth leads to the expansion of the wildland‐urban interface, with emissions from prescribed burning around the HMA adding to urban pollution (Nowak & Walton, 2005).

Prior studies have examined UHI and UPI associated with the HMA (Moser et al., 2017; Streutker, 2002, 2003). However, the impact of the last two decades' population and urban land cover expansion on the UHI, UPI, social vulnerability, and their interactions remain unexamined. A major hurdle is the inadequacy of sparse meteorological and air quality networks for monitoring UHI and UPI growth. This concern is heightened by the HMA's complex spatial pollution variability, influenced by approximately 400 chemical manufacturing units, two of the U.S.'s biggest oil refineries, daily vehicular traffic exceeding 140 million miles a day, small urban sources such as gas stations, restaurants, dry cleaners, and construction, and episodic smoke transport from fires in Mexico, Central America, and surrounding states (O’Dell et al., 2020; Sexton & Linder, 2015; Sexton et al., 2007). Notably, pollution sensors on Google Street View cars in the HMA reveal fine‐scale maxima of air pollution undetected by traditional air quality monitoring networks (Miller et al., 2020).

Implications of Miller et al. (2020) are quite substantial, as the United Nations (2022) projects that by 2050, 87% of the American population and 68% of the world population will live in urbanized locations. This study thus addresses the above mentioned limitations by utilizing the National Aeronautical Space Administration (NASA) Terra and Aqua Earth‐observing platforms' aerosol and land temperature products which are combined with situ observations and socioeconomic data sets to quantify the UHI and UPI (specifically particulate pollution) growth over the HMA. This study aims to: (a) characterize urban growth that has occurred over the HMA during the last two decades, (b) characterize associated growth of the UHI and UPI, and (c) analyze how the combined effects of the UHI and UPI contribute to environmental inequalities in the region.

## 2 Study Area

The U.S. census‐designated area of Houston ‐The Woodlands‐Sugarland Metropolitan Area (HMA) is situated in southeastern Texas along the Gulf of Mexico (GoM). It includes Harris County, containing the city of Houston, and the surrounding counties—Galveston, Chambers, Liberty, Montgomery, Waller, Austin, Fort Bend, and Brazoria. The HMA approximately occupies 24,459 km^2^, surpassing the size of New Jersey. Houston, the fourth most populous U.S. city, had a city‐limits population of nearly 2.3 million and a metropolitan area population of 6.5 million as of 2021 (United Nations, 2022; U.S. Census Bureau, 2022).

## 3 Data and Methodology

The data sets and analysis methodology used in this study are described in Sections 3.1, 3.2, 3.3, 3.4, 3.5, 3.6.

### 3.1 Satellite Observations

To quantify multi‐decadal urban land cover growth in the HMA, we utilize the National Land Cover Database (NLCD) land cover classification for the contiguous U.S. (CONUS). Derived from Landsat satellite observations at 30 m spatial resolution, (C. G. Homer et al., 2007; C. H. Homer et al., 2012; C. Homer et al., 2015) NLCD is available at 2–3‐year intervals from 2001 to 2021. NLCD classifies locations on a 30 m CONUS grid into one of 34 land cover classes, with high‐density, medium‐density, low‐density, and open‐space urban classes relevant to this study. Our analysis uses the 2001 and 2019 NLCD data sets to examine and quantify multi‐decadal changes in spatial patterns and the extent of urban land cover.

We analyze alterations in Land Surface Temperature (LST), vegetation cover, and atmospheric aerosol loading using land and aerosol products derived from the Moderate Resolution Imaging Spectroradiometer (MODIS) sensor on NASA’s Terra and Aqua platforms. Both satellites follow sun‐synchronous orbits, passing over the HMA daily at around 10:30 a.m. and 1:30 p.m. local time. For consistency and data availability throughout the first two decades (Aqua operations commenced midway through 2002) and to minimize cloud contamination, we only utilized land products from Terra. The eight‐day composite day and night LST (MOD11A2) at 1 km spatial resolution with retrieval errors of 2.0 ± 0.5 K (Wan, 2014) is used to investigate UHI changes. Changes in urban greenness were examined using the 16‐day composite Normalized Difference Vegetation Index (NDVI) product at 250 m spatial resolution (MOD13Q1). NDVI is calculated as the ratio of the difference between near‐infrared and red reflectance channels normalized by the sum of reflectance values of the same channels. With values ranging from −1 to 1, NDVI signifies extremes of bare ground (zero green vegetation cover) and full coverage by green vegetation at a given location (Didan et al., 2015).

The Aerosol Optical Depth (AOD) product utilized is a daily 0.55 μm Terra and Aqua composite with 1 km spatial resolution, (MCD19A2). The AOD is calculated using the multi‐angle implementation of the atmospheric correction (MAIAC) algorithm (Lyapustin & Wang, 2018) and reflects the total aerosol mass within the atmospheric column over a specific location. To ensure data consistency and avoid potential anomalies from Aqua observations available only beginning in mid‐2002, the AOD analysis focuses solely on data from 2003 to 2019.

All of the satellite data were accessed and analyzed using Google Earth Engine (GEE). Note that GEE is a global cloud‐computing platform that provides access to satellite imagery and derived products acquired over the last 50 years (Gorelick et al., 2017).

### 3.2 Surface Meteorological and Air Quality Observations

We analyzed surface temperature, humidity, and particulate pollution trends in the HMA using in situ meteorological and air quality observations. Ten meteorological sites‐ four in the Houston city limits, three in the suburbs, and three on the outskirts‐ were also used for statistical regression modeling to develop high‐resolution spatial patterns of monthly maximum air temperature. Data were obtained from NOAA’s National Centers for Environmental Information (NCEI), with the station details in Table S5 of the Supporting Information S1, ensuring consistency in station locations.

For particulate air quality, PM2.5 concentrations from six EPA monitoring stations within the HMA were used. PM2.5 refers to inhalable particulates with a diameter of less than 2.5 μm, posing the greatest health risk.

### 3.3 Social Vulnerability Index

Social vulnerability, encompassing socioeconomic and demographic factors affecting community resilience to external stresses, (Flanagan et al., 2020; Mah et al., 2023) can be quantified through a social vulnerability index (SVI). The CDC’s SVI data set, derived from U.S. Census Bureau's census tract data, incorporates various factors such as socioeconomic status, household composition, disability, minority status, housing, and transportation attributes (Flanagan et al., 2020). The overall SVI ranking used here represents the nationwide percentile ranking of social vulnerability for a census tract. An SVI value of 1 indicates that all other census tracts have equal or lower social vulnerability, while a value of 0 means none have equal or lower social vulnerability compared to the considered census tract. This study employs SVI to assess the societal impacts of the UHI and UPI in terms of environmental inequalities.

### 3.4 Satellite Data Analysis of Urban Growth, LST, NDVI, and AOD

We analyzed NLCD LULC classifications for 2001 and 2019, quantifying transitions between high, medium, low‐density, and open‐space urban classes. Urban land cover masks for both years were used to calculate statistics for LST, NDVI, AOD, SVI, maximum air temperature, and PM2.5 separately in urban and non‐urban regions. To study the influence of urban growth, a mask identifying changes in urban extent between 2001 and 2019 was derived.

To assess changes in LST, NDVI, and AOD, we generated decadal average spatial maps for 2000–2009 and 2010–2019. This involved the computation of monthly averages of each variable (${\bar{\rho }}_{m}\left(\lambda ,\phi \right)$) for each pixel across the HMA domain:

(1) $${\bar{\rho }}_{m}\left(\lambda ,\phi \right)=\frac{\sum_{i=1}^{N}\rho \left(xy,i\right)}{N}$$

where *ρ*(*λ*, *ϕ*, *i*) is the gridded satellite‐derived value of the variable at a given latitude (*x*) and longitude (*y*), *i* is a specific instance of observation/retrieval, and *N* the total number of instances. Subscript *m* refers to the monthly mean of the variable. The variable average (${\bar{\rho }}_{d}\left(\lambda ,\phi \right)$) for the first and second decades, where *d* refers to the decadal mean, is then computed by averaging the monthly averages for the periods of 2000–2009 and 2010–2019, except for AOD. For the first decade, the AOD average is computed using 2003–2009 data to account for the unavailability of Aqua observations before mid‐2002. Note that we utilized decadal average spatial maps, as only persistent features will be retained in long‐term averages, thereby increasing the confidence in change analysis.

To quantify temporal changes in urban regions, we spatially averaged decadal mean maps of day and night LST, NDVI, and AOD over census tracts in the HMA region with more than 50% urban land cover (referred to as urban census tracts). Urban census tracts for the 2000–2009 period were defined using 2000 census tracts and 2001 NLCD classifications, while those for the 2010–2019 period used 2020 census tracts and 2019 NLCD classifications. Trend analysis for the 2000–2019 period is conducted using time series created from spatial averages of monthly mean LST, NDVI, and AOD maps for urban census tracts. The 2020 census tracts and 2019 NLCD classification were used for this computation to ensure a consistent average for meaningful trend analysis. The seasonal Mann‐Kendall test determined statistically significant monotonic trends, and the Thiel‐Sen slope quantified the trend (Kendall, 1948; Mann, 1945; Sen, 1968; Thiel, 1950). Note that for the processing of LST for computing decadal averages and time series, the methodology of L. Hu and Brunsell (2013) was applied to minimize cloud contamination in our results.

### 3.5 Analysis of Surface Meteorological and Air Pollution Observations

To link satellite analysis of LST with near‐surface air temperature and particulate pollution, we performed a time series analysis of monthly averaged values for daily maximum air temperature and PM2.5 concentrations. The time series was examined for monotonic trends using the Mann‐Kendall seasonal test, and the trends were quantified using the Sen slope.

We employed statistical regression modeling to fuse surface observations with satellite data, creating high‐resolution estimates of monthly average maximum air temperature. Multiple linear regression models were developed for monthly average maximum air temperature (*T*_max_), using the following predictors chosen based on prior research (de Souza et al., 2022; Yuvaraj, 2020): monthly average satellite‐derived LST and season (expressed categorically as DJF‐1, MAM‐2, JJA‐3, SON‐4). Regression modeling utilized monthly averaged maximum temperature from 10 meteorological stations, paired with corresponding monthly averaged daytime LST. The model was trained on 80% of the data and tested on the remaining 20%. The *T*_max_ model was then applied to generate high‐resolution maps of decadal seasonal average maximum air temperatures for the first and second decades and analyze the impact of urban growth.

We utilized high spatial resolution maps of surface PM2.5 derived from a data fusion approach. Instead of developing models, we employed the surface PM2.5 data set of Van Donkelaar et al. (2021), which integrates MAIAC AOD, other NASA aerosol products, surface observations, and chemical transport model outputs. This validated data set offers monthly average PM2.5 maps (PM_avg_), enabling the estimation of changes in PM2.5 concentrations due to urban growth.

### 3.6 Social Vulnerability Analysis

We compared SVI spatial patterns between 2000 and 2020 to assess how urban growth affects the social vulnerability of the HMA. Census tracts were grouped into four categories based on SVI values (0.0–0.25, 0.25–0.50, 0.50–0.75, and 0.75–1.0). For each group, decadal average LST, NDVI, AOD, maximum air temperature, and surface PM2.5 were computed to evaluate the combined effects of UHI, UPI, and social vulnerability.

## 4 Results

### 4.1 Multi‐Decadal LULC Change Impacts in the Houston Metropolitan Area

Between 2001 and 2019, the HMA witnessed a substantial 1,345 km^2^ (22.6%) increase in urban land cover (Figure 2, Table 1), equivalent to adding two times the entire urban area of New York City. This growth primarily resulted from converting agricultural and woodland classes to urban and built‐up land cover. Medium density urban land cover showed the highest growth in both area and percentage, followed by high‐density urban land cover, while the open space urban category exhibited the least growth. The spatial pattern of urban growth expanded in all directions, with higher growth in the northern, northwestern, and southwestern directions. The HMA consistently experienced positive urban growth, with annual percent increases ranging from 1.8% to 5.1% between 2001 and 2019, reflecting a steady yet occasionally accelerated urbanization trend.

**Figure 1:**
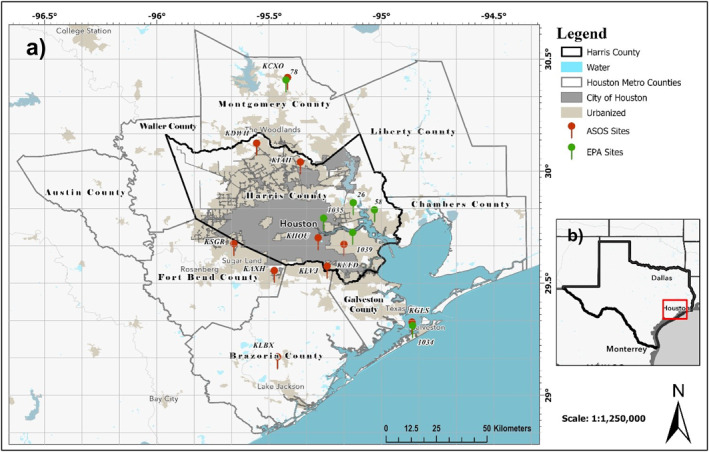
(a) The spatial extent of the study area for this paper, with the red box in the inset (b) showing the location of the study area within the state of Texas.

**Figure 2:**
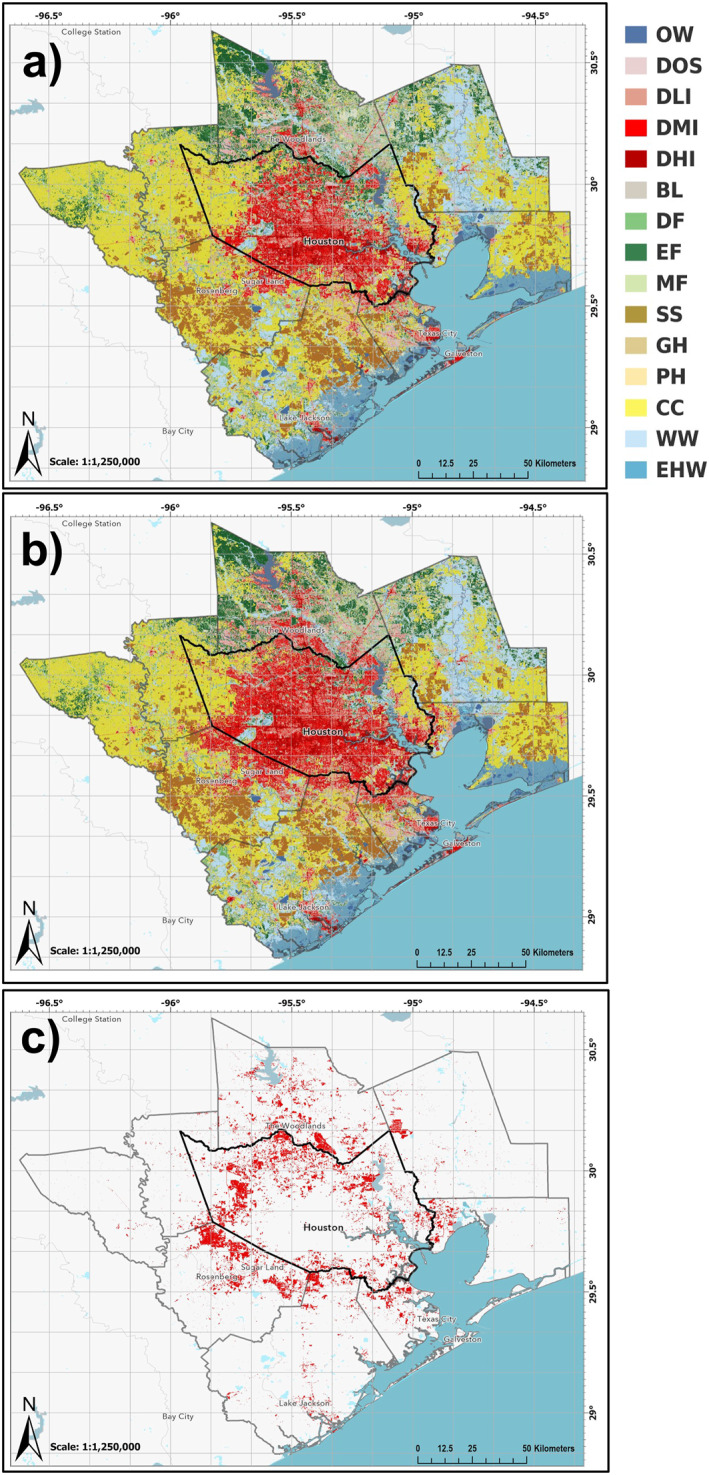
2001 (a) and 2019 (b) NLCD LULC across the HMA, and the new urbanization footprint (in red) from 2001 to 2019 (c). Reds in (a) and (b) indicate urbanized classifications, where DLI, DMI, and DHI are low, medium, and high intensity urban respectively. The complete list of abbreviation meanings can be found in Supporting Information S1.

**Table 1:** Mean Values of Selected MODIS Variables As Well As Surface *T*_max_ and PM2.5 Across the HMA Urban Census Tracts, Isolated by SVI Bins

	2000–2009 (2010–2019)						2010s–2000s difference					
SVI bin	LST day	LST night	NDVI	AOD	*T*_max_	PM_2.5_	LST day	LST night	NDVI	AOD	*T*_max_	PM_2.5_
0.0–0.25	300.48 (300.97)	289.83 (289.94)	0.49 (0.49)	0.15 (0.14)	304.71 (304.99)	11.39 (9.02)	1.023	0.456	−0.021	−0.011	0.407	−2.323
0.25–0.50	300.12 (300.78)	289.40 (289.80)	0.52 (0.50)	0.14 (0.14)	304.37 (304.66)	11.03 (8.72)	0.816	0.404	−0.018	−0.005	0.341	−2.306
0.50–0.75	300.39 (300.92)	289.73 (289.84)	0.49 (0.49)	0.15 (0.14)	304.61 (304.74)	11.25 (8.73)	0.750	0.370	−0.016	−0.010	0.318	−2.298
0.75–1.0	300.84 (301.83)	289.84 (290.28)	0.46 (0.45)	0.15 (0.15)	304.83 (305.41)	11.30 (9.36)	0.837	0.344	−0.019	−0.002	0.327	−2.236

**Figure 3:**
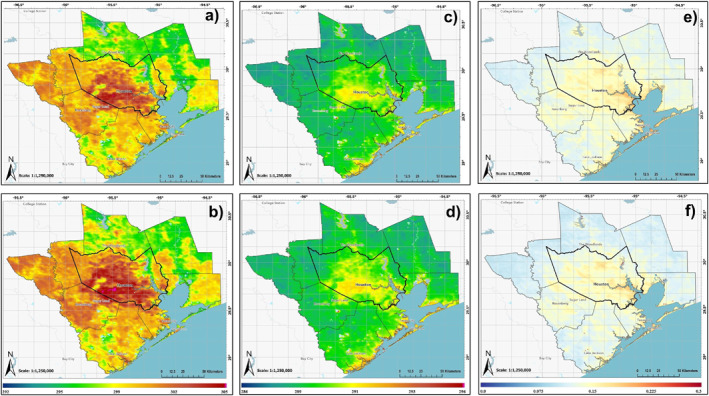
Decadal‐averaged spatial distributions of MODIS‐derived monthly mean Daytime LST (a, b), Nighttime LST (c, d), and AOD (e, f). The 2000–2009 decade is displayed in the top row, and the 2010–2019 decade is displayed in the bottom row. Note that AOD data begins in 2002, while LST Day and Night both extend back to 2000.

**Figure 4:**
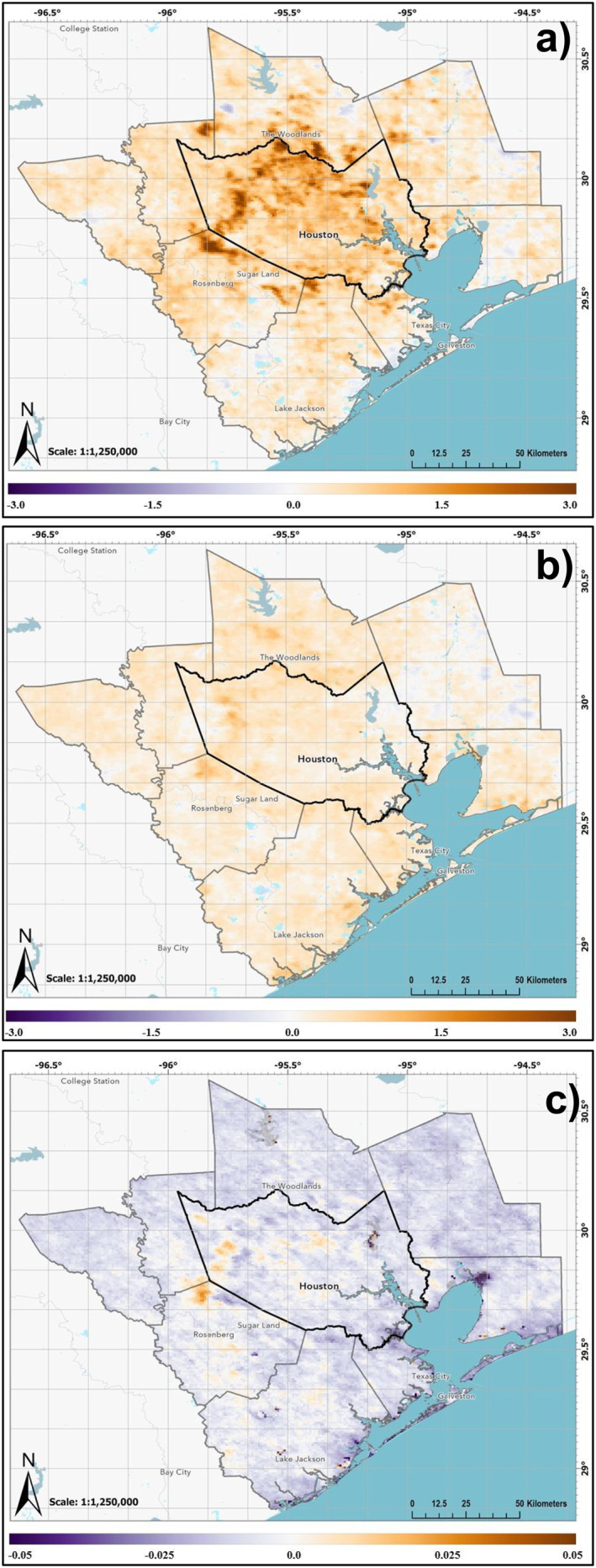
(a) Daytime LST, (b) Nighttime LST, and (c) AOD 2010s decadal average minus 2000s decadal average over the HMA.

Figure 3 shows decadal averages of daytime and nighttime LST as well as AOD. Spatial patterns of daytime and nighttime LST align with urban land cover growth in Figures 2c and 3a–3d, exhibiting higher values over urban areas. Decadal‐averaged daytime LST for urban majority census tracks surpassed non‐urban tracts during both 2000–2009 and 2010–2019 (Table S3 in Supporting Information S1). The contrast in decadal average daytime (nighttime) LST between urban and non‐urban tracts increased from ∼4.2 (1.8) K to 4.5 (1.9) K between the two decades. Urban tracks’ decadal‐averaged daytime (nighttime) LST increased by ∼0.86 (0.40) K from 2000 to 2009 to 2010–2019, with a mean difference of ∼0.30 (∼0.13). We used the decadal average daytime LST for urban census tracts during the 2000–2009 period as a threshold (∼303 K) to define the urban LST footprint. Using this threshold, the daytime urban LST footprint expanded from 527 km^2^ (2000–2009) to 1,433 km^2^ (2010–2019). Figure 4 reveals the spatial difference in decadal average AOD and daytime and nighttime LST between the 2010–2019 and 2000–2009 periods. Both the day and night LST difference patterns align with the pattern of differences in urban land cover between the two decades (Figure 2c), although less pronounced in the case of nighttime LST. Decadal average analysis of NDVI and AOD also reveals differences in mean values between urban and non‐urban regions (Table S3 in Supporting Information S1). Decadal average NDVI for non‐urban census tracts remains stable between 2000–2009 and 2010–2019, but urban census tracts show a decrease of ∼0.006. The spatial pattern of NDVI changes shows maximum differences (Figure S3 in Supporting Information S1) in areas of urban land cover growth (Figure 2c). Decadal average AOD also show higher mean values over urban census tracts compared to non‐urban tracts in both decades. However, the decrease in mean decadal AOD between the two decades is less for urban census tracts than for non‐urban tracts. While a mean difference in AOD of −0.004 is found in the newly urbanized regions, the spatial pattern of decadal average AOD do show small, localized increases in pockets over such areas (Figure 4c).

Utilizing the seasonal Mann‐Kendall test, we assessed statistically significant monotonic trends in time series of monthly mean daytime and nighttime LST, NDVI, and AOD spatially averaged for urban census tracts (Figure S2 in Supporting Information S1). For the 2000–2009 period, statistically significant monotonic trends were observed only for NDVI and nighttime LST when analyzed separately for each decade (Table S4 in Supporting Information S1). However, when considering the entire multi‐decadal period, all variables displayed statistically significant monotonic trends. Daytime and nighttime LST exhibited increasing trends, with the magnitude of Sen slope indicating a higher daytime trend compared to nighttime. In contrast, both NDVI and AOD showed decreasing trends over the multi‐decadal study period.

### 4.2 Impact of Urbanization on Daytime Patterns of Near‐Surface Air Temperature and PM2.5

We used in situ observations to assess if trends in satellite‐derived LST and AOD correspond to changes in near‐surface air temperature and PM2.5 concentrations (Table S6, Figure S4 in Supporting Information S1). Therefore, we examined time series trends of maximum and minimum air temperatures and average PM2.5 concentration observations and how they vary spatially.

We found that most stations exhibited statistically significant, monotonically increasing trends in maximum and minimum surface air temperature (Table S6 in Supporting Information S1). Minimum temperature trends generally exceeded maximum temperature trends, with higher values observed in regions of urban growth. Urban stations also showed greater trends, with maximum temperature trends higher by ∼0.013 K year^−1^ and minimum temperature trends higher by ∼0.040 K year^−1^. The Galveston (KGLS) and Hooks Memorial Airport (KDWH) localities, coinciding with observed urban growth, displayed the largest trends in maximum and minimum temperatures, respectively. Houston Hobby Int’l Airport (KHOU), within the urban core with minimal land cover changes, exhibited lower Sen slope estimates.

All three PM2.5 monitoring stations that reported at least 10 years of continuous data (EPA stations 482010058, 482011039, and 482011035, where 48201 is the code for Harris County, Texas; Figure 1) exhibited statistically significant decreasing trends, indicating a general reduction in particulate pollution. Station 0058, surrounded by the least impervious surface, showed the lowest trend magnitude, while station 1035, within the highest impervious surface, displayed the highest trend. The spatial pattern of these trends reflects an overall reduction in particulate pollution influenced by Clean Air Act regulations, which has been shown in prior studies (Environmental Protection Agency, 2019). However, localized urban pollution sources weaken the effectiveness of these regulations, and this effectiveness also varies based on the degree of urbanization. This limited analysis, in line with spatial patterns in decadal average AOD (Figures 3e, 3f, and 4c), underscores the necessity for a more extensive surface PM2.5 network for broader validation.

**Figure 5:**
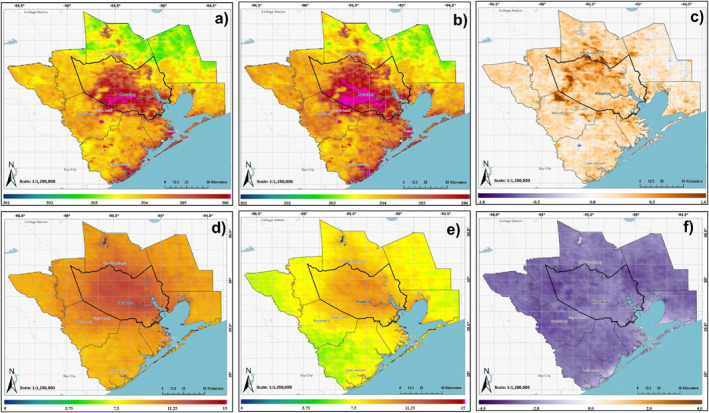
Decadal‐averaged spatial distributions of predicted *T*_max_ (a, b), produced from multi‐variate ordinary least squares linear regression and reported in K, and surface PM2.5 (d, e). Decadal differences of *T*_max_ (c) and PM2.5 (f) are also displayed.

We employed a multiple linear regression model for monthly average maximum air temperature (*T*_max_) to infer high‐resolution spatial patterns. The constructed *T*_max_ model (Table S7 in Supporting Information S1) yielded an R^2^ which value of 0.844 and an RMSE of 1.62 K, demonstrating performance comparable to or better than similar models in prior studies (de Souza et al., 2022; Yuvaraj, 2020). Utilizing the *T*_max_ model, we generated monthly maps of average maximum temperature for the HMA region. These maps were then used to compute decadal average seasonal and annual maximum temperature maps for both the 2000–2009 and 2010–2019 decades (Figure 5).

Spatial maps of maximum temperature reveal contrasts between urban regions and their surroundings, intensifying during the second decade. The difference in decadal average maximum temperature between the two decades (Figure 5) highlights a robust warming signal along the ring of new urbanization from 2001 to 2019 (Figure 2c), aligning with earlier time series trend analysis. Although decadal differences can exceed 2 K in specific locations, the mean difference in decadal average maximum temperature for urban regions is approximately ∼0.25 K. This enhanced warming signal associated with urbanization is present throughout all seasons (Figure S6 in Supporting Information S1), with the most notable contrast occurring during the spring season.

We conducted a similar analysis using downscaled surface PM2.5, combining in situ observations with satellite data sets. Despite the absence of a notable trend in AOD (Tables S3 and S4 in Supporting Information S1), in situ sensors indicated some decreasing trends in PM2.5 (Figure S5 in Supporting Information S1). While the downscaled analysis was expected to mirror the trends observed by in situ sensors, our findings reveal contrasting implications. First, decadal average patterns of downscaled PM2.5 show higher values over urban regions compared to the surroundings. Mean PM2.5 concentrations in urban locations exceeded the primary standards set by the EPA (12 µgm^−3^) in the first decade but not in the second decade. Differences in decadal average surface PM2.5 indicate the least change in concentrations along the ring of new urbanization throughout the study period (Figures 2c and 5f). Notably, the decadal difference in PM2.5 falls below −3 µgm^−3^ at certain locations, but the mean decadal difference is −2.34 µgm^−3^. Second, this discrepancy between AOD and PM2.5 trends suggests that column‐total AOD, which includes contributions from atmospheric transport, has different controlling factors compared to in situ observations. Thus, PM2.5 trends may be more reflective of the ongoing urbanization process as they include less transport from other regions.

### 4.3 Socioeconomic Impacts of Rapid Urbanization

**Figure 6:**
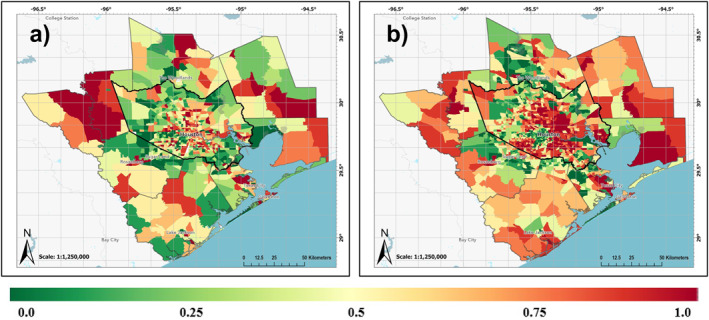
2000 (a) and 2020 (b) SVI ranking (0‐1) for HMA census tracts.

Finally, we assessed changes in social vulnerability within the HMA over the multi‐decadal study period and investigated how interactions between heat, pollution, and social vulnerabilities contribute to environmental inequalities. Census tracts were categorized based on SVI percentile rankings: 0.0–0.25 (least socially vulnerable), 0.25–0.50, 0.50–0.75, and 0.75–1.0 (most socially vulnerable). The spatial distribution of the SVI revealed a significant increase in social vulnerability within the urban regions of the HMA in the last two decades (Figure 6). The largest driver of this change in the main urban core of the HMA was found to be socioeconomic status, followed closely by household characteristics. The most socially vulnerable urban census tracts in the HMA increased by 629 tracts during this period.

Spatial patterns of decadal average day and nighttime LST, AOD, maximum surface air temperature, and surface PM2.5 concentrations reveal census tracts with the highest SVI are situated in areas where populations are most susceptible to heat and pollution exposure (Figures 3 and 5). The mean of decadal average day and nighttime LST, AOD, maximum air temperature, and PM2.5 indicates higher values for the most vulnerable census tracts during both decades (Table 1).

The change in LST and surface air temperature between the two decades is most significant for the least socially vulnerable census tracts, possibly due to increased urbanization in suburban regions of Houston (Figure 2c). Although NDVI also shows the highest decrease for the least vulnerable census tracts, the mean decadal average NDVI is lowest for census tracts with the most social vulnerability, suggesting reduced access to green spaces in these areas. Mean decadal averages of both AOD and surface PM2.5 indicate a decrease in particulate matter air quality across all census tract categories. However, the decrease between the two decades is least for the most socially vulnerable census tracts and higher for census tracts with the least social vulnerability. Although this analysis was conducted for specific SVI bins across the entire HMA, focusing on a newly urbanized census tract to another with identical vulnerability but no urbanization, the newly urbanized tract experiences the most significant increase in heat stress.

## 5 Uncertainty and Limitations

The following methodological limitations need to be considered in the interpretation of the study findings. In addition to errors inherent in the retrieval of MODIS land and aerosol products, retrieval is only valid under cloud‐free conditions. While long‐term averaging mitigates the impact of retrieval errors, cloud cover modulates the number of valid retrievals at any given geographical location. Thus, the number of retrievals used to construct decadal averages varies at each geographical location. Further, spatial variability of cloud cover itself can be affected by urban growth (Theeuwes et al., 2019; Vo et al., 2023), thereby impacting computed statistics of the variables utilized in the study (and thus spatial patterns in decadal averages and trend analysis). Given these limitations of the study, future work may include relatively high‐resolution numerical model experiments incorporating scenarios of realistic LULC changes over the multidecadal period.

## 6 Discussion and Conclusions

Our analysis shows that the HMA, one of the largest metropolitan areas in the United States, has experienced rapid urbanization over the past two decades, adding ∼1,345 km^2^ of developed land cover, which is nearly twice as large as the area of New York City. This urban growth is accompanied by both UHI and UPI growth as well as heightened social vulnerability. We found that both the day and nighttime UHI LST footprint also expanded with urban growth and the decadal average values showed increases even within regions that were urbanized before the study period, with corresponding increases in UHI as reflected by increases in maximum and minimum air temperatures (Table 1, Table S3 in Supporting Information S1).

While the UPI associated with the HMA experienced an overall reduction in particulate pollution, the magnitude of the decrease is less when compared to rural areas. Over the urban areas, decadal changes in AOD and surface PM2.5 both show higher spatial variability compared to the rural surrounding areas, and exhibit a more homogenous and drastic reduction during the second decade of the study. While our analysis shows that the Clean Air Act of 1990, with subsequent evaluations having occurred most recently in 2012, resulted in an overall decrease in particulate pollution within the UPI. However, the increased volume of vehicular traffic (Figure S7 in Supporting Information S1) and other sources associated with urban growth appear to locally counteract the overall reduction of emissions from individual sources. These findings align with similar studies (Lim et al., 2020; Miller et al., 2020; Southerland et al., 2022), specifically Miller et al. (2020), which found local pockets of air pollution within the HMA that are not detected by existing sparse‐density air quality networks. Our finding that the co‐occurrence of such features with localized maxima of urban heat and social vulnerability highlights the necessity for advanced data fusion methodologies that incorporate satellite data, numerical modeling, and low‐cost sensor inputs for urban heat and gaseous pollution mapping (Pochwała et al., 2020; Sulzer et al., 2022).

We find synergies arising from population growth, heightened social vulnerability, UHI expansion, and localized PM2.5 increases within the UPI exacerbate environmental inequalities in the HMA, with implications amid climate change concerns. A recent study links approximately 700 yearly deaths to nationwide heat stress (Vaidyanathan et al., 2004), and since Anderson and Bell (2011) report ∼2.5% mortality risk rises per 1°F (∼0.55°C) heatwave intensity, heat stress‐induced deaths may increase as heatwaves are projected to occur more frequently (Meehl & Tebaldi, 2004; Perkins et al., 2012).

Our regression analysis indicates that urban growth may amplify heatwave events in the HMA by an additional 0.55 K, elevating risks. Combined exposure to heat stress and particulate pollution can result in a 250% mortality risk increase from individual effects (Analitis et al., 2018; X. Hu et al., 2022; Stafoggia et al., 2023). Our analysis reveals urban growth contributes to localized particulate pollution. Numerous studies highlight higher risks for socially vulnerable populations from heat stress and particulate pollution (Khatana et al., 2022; O’Lenick et al., 2019). Thus, our findings of increased local social vulnerability with urban growth, alongside heightened heat stress and pollution, are concerning, especially with the projected increase in heatwave events, as was observed in the summer of 2023.

The United Nations (2022) projection that 87% of all Americans and 68% of the world population will live in urban areas by 2050 provides the perspective for considering the local and broader implications of our study. Further, the EPA Integrated Climate and Land Use Scenarios (ICLUS) data set suggests that the urban land cover within the HMA will continue expanding during the upcoming decades. Unless steps are taken to mitigate the UHI and UPI effects, their footprints will likely grow also. Therefore, if the observed relationships between urban social vulnerability, heat stress, and air quality continue, environmental inequalities will be exacerbated.

Our finding that environmental inequalities are worsened by the combined effects of urban heat islands (UHI), urban pollution islands (UPI), and social vulnerability is supported by prior studies (Y. Li et al., 2020; Nair et al., 2023; Sabrin et al., 2020; Tang et al., 2017) and is relevant to other U.S. cities and megacities worldwide. We also found that HMA did not experience increase in green spaces that mitigate UHI (Figure S3c in Supporting Information S1), highlighting the need of adopting such UHI mitigation strategies.

Our study demonstrates the utility of data fusion, incorporating satellite observations for high‐resolution mapping of the UHI and UPI to identify environmental inequalities and associated human health risks. This data fusion approach can help in the formulation of policies for mitigation and can easily be applied to other cities experiencing comparable urban growth.

## Supporting Information

The supplemental information to this study, with figures and tables denoted with a leading “S” in the manuscript text when referenced, are provided in a separate document.

## Conflict of Interest

The authors declare no conflicts of interest relevant to this study.

## Supporting information

Supporting Information S1

## Data Availability

All data sets utilized for the analysis presented in this manuscript are publicly available. Software utilized for data analysis are Python, R, ArcGIS Pro, and Google Earth Engine (Gorelick et al., 2017). The land cover data was sourced from the National Land Cover Database (NLCD) for the years 2001 and 2019 (C. G. Homer et al., 2007; C. H. Homer et al., 2012; C. Homer et al., 2015). Land surface temperature, vegetation cover, and aerosol optical depth data were derived from MODIS products, specifically MOD11A2, MOD13Q1, and MCD19A2 (Didan et al., 2015; Lyapustin & Wang, 2018; Wan, 2014). Surface meteorological data was obtained from NOAA’s National Centers for Environmental Information (NCEI), and PM2.5 data was collected from EPA monitoring stations. Social vulnerability was assessed using the CDC’s Social Vulnerability Index (SVI) (Flanagan et al., 2020; Mah et al., 2023). Surface PM2.5 data was further analyzed using the data set provided by Van Donkelaar et al. (2021).
