# Supplementary material for: Synergy of Urban Heat, Pollution, and Social Vulnerability in One of America's Most Rapidly Growing Cities: Houston, We Have a Problem
Source: Geohealth. 2024 Sep 4;8(9):e2024GH001079. doi: 10.1029/2024GH001079 (PMC11372823; doi:10.1029/2024GH001079)
Supplement: Supplementary file 1 — Supporting Information S1 [file GH2-8-e2024GH001079-s001.pdf]

Supplemental Material for:

Synergy of Urban Heat, Pollution, and Social Vulnerability in One of America's Most Rapidly Growing Cities: Houston, We Have a Problem

Andrew Blackford[1], Trent Cowan[1], Udaysankar Nair[1], Christopher Phillips[2], Aaron Kaulfus[3], Brian Freitag[3]

[1]Department of Atmospheric and Earth Science, The University of Alabama in Huntsville, Huntsville, AL, USA.

[2]Earth System Science Center, The University of Alabama in Huntsville, Huntsville, AL, USA.

[3]National Aeronautical and Space Administration, Marshall Space Flight Center, Huntsville, AL, USA.

Table of Contents

NLCD LULC Classification Abbreviation Meanings pg. 3

Supplemental Tables pg. 4

Supplemental Figures pg. 9

NLCD LULC Classification Abbreviation Meanings

OW	Open Water
DOS	Developed Open Space
DLI	Developed Low Intensity
DMI	Developed Medium Intensity
DHI	Developed High Intensity
BL	Barren Land
DF	Deciduous Forest
EF	Evergreen Forest
MF	Mixed Forest
SS	Shrub/Scrub
GH	Grassland/Herbaceous
PH	Pasture/Hay
CC	Cultivated Crops
WW	Woody Wetlands
EHW	Emergent Woody Wetlands
PIS	Perennial Ice/Snow

Descriptions of the classifications can be found at https://www.mrlc.gov/data/legends/national-land-cover-database-class-legend-and-description

Table S1. Urbanized LULC changes between 2001 and 2019, using NLCD classifications for the Houston metropolitan area. Spatial values are reported in km^2.

LULC Classification	2001 Classification	2019 Classification	LULC Lost	LULC Gained	Net LULC Change	Percent Change
High Density Urban	676.53	974.17	-0.10	294.97	297.64	30.55
Medium Density Urban	1324.24	1932.37	-9.32	617.38	608.13	31.47
Low Density Urban	1363.47	1614.42	-111.37	362.31	250.94	15.54
Open Space Urban	1352.08	1441.30	-205.08	294.27	89.23	6.19
**Total**	**4617.18**	**5962.26**	**-325.87**	**1568.94**	**1345.09**	**22.56**

Table S2. Area of NLCD classifications changed to urbanized classes from 2001-2019, broken up by each county in the HMA.

NLCD Class	HARRIS	GALVESTON	CHAMBERS	LIBERTY	MONTGOMERY	WALLER	AUSTIN	FORT BEND	BRAZORIA
**CC**	5.24	4.88	1.22	0.23	0.00	3.14	0.23	36.79	11.67
**PH**	219.88	17.97	28.63	8.28	23.30	14.96	4.45	135.74	41.28
**GH**	26.20	7.96	0.72	5.99	22.99	0.44	0.03	9.98	8.78
**DF**	28.38	9.01	1.76	0.46	1.11	0.26	0.07	16.90	9.42
**EF**	109.66	3.19	0.52	10.91	58.71	1.17	0.10	2.35	2.53
**MF**	39.44	2.26	3.39	10.27	65.29	0.60	0.00	4.50	3.29
**SS**	25.56	2.72	0.26	0.57	4.76	0.31	0.03	13.63	3.02
**WW**	46.48	4.63	5.54	5.66	19.69	0.10	0.03	16.04	7.50
**EHW**	12.29	5.31	1.29	0.80	1.29	0.05	0.00	3.70	7.85
**BL**	11.12	0.90	0.28	0.18	1.89	0.31	0.05	4.07	1.49
**OW**	26.42	1.47	0.39	0.05	0.78	0.05	0.03	0.93	1.11
**PIS**	0.00	0.00	0.00	0.00	0.00	0.00	0.00	0.00	0.00
**TOTAL**	**550.67**	**60.30**	**44.00**	**43.40**	**199.81**	**21.39**	**5.02**	**244.63**	**97.94**

Table S3. Maximum, Minimum, and Mean values for selected MODIS products over NLCD majority urban (non-urban) pixels over the HMA, from 2000-2019 (2002-2019 for AOD, see Section 3.5). The decadal difference section reports maximum increase, decrease, and mean difference using 2010-2019 minus 2000-2009.

MODIS PRODUCT	2000-2009			2010-2019			Decadal Difference		
	Max.	Min.	Mean	Max.	Min.	Mean	Max.	Min.	Mean
LST Day (K)	304.52 (304.49)	300.77 (292.24)	303.26 (298.67)	305.41 (304.80)	303.16 (291.94)	304.10 (299.09)	4.31 (3.82)	-0.68 (-1.08)	0.86 (0.42)
LST Night (K)	291.40 (295.76)	289.39 (287.09)	290.70 (288.73)	291.73 (295.51)	290.23 (287.44)	291.14 (289.04)	1.75 (3.49)	-0.72 (-1.20)	0.40 (0.31)
NDVI	0.68 (0.82)	0.24 (-0.20)	0.39 (0.58)	0.67 (0.82)	0.25 (-0.20)	0.39 (0.59)	0.24 (0.65)	-0.52 (-0.60)	-0.02 (0.001)
AOD	0.22 (0.31)	0.10 (0.06)	0.14 (0.14)	0.47 (0.58)	0.00 (0.00)	0.14 (0.13)	0.30 (0.34)	-0.21 (-0.19)	-0.007 (-0.01)

Table S4. Seasonal Theil-Sen slope and Mann Kendall analyses of the four MODIS-derived variables examined, subset to urban census tracts in the HMA, for the period 2000-2019 (AOD 2002-2019) broken out by decade. Sen slopes are reported in per year in this table.

DECADE:	**2000-2009**			**2010-2019**			**Whole Period (2000-2019)**		
**Variable**	**Sen Slope**	**P Value**	**Tau**	**Sen Slope**	**P Value**	**Tau**	**Sen Slope**	**P Value**	**Tau**
**Day LST**	-0.055	0.130	0.111	0.034	0.737	-0.026	0.0729	7.0187e-5	0.1871
**Night LST**	-0.036	0.007	0.198	0.050	0.661	0.033	0.0674	1.2716e-6	0.2296
**NDVI**	-0.00018	2.31e-8	-0.405	0.00025	0.115	0.115	-0.0019	4.4694e-10	-0.2932
**AOD**	-0.000013	0.574	-0.024	-0.000014	0.833	-0.004	-0.0001	0.4237	-0.0131

Table S5. Information on the ten selected surface observing stations in the HMA chosen for this study.

ICAO	Latitude	Longitude	Elevation (m)	Start Year	End Year
KIAH	29.984	-95.361	27.5	2002	2022
KGLS	29.270	-94.864	1.5	2005	2022
KHOU	29.646	-95.282	13.2	2005	2022
KEFD	29.617	-95.167	9.8	2005	2022
KLVJ	29.519	-95.242	11.9	2006	2022
KSGR	29.620	-95.657	23.5	2006	2022
KCXO	30.361	-95.418	69.4	2006	2022
KDWH	30.068	-95.556	46.8	2006	2022
KAXH	29.500	-95.477	21.0	2009	2022
KLBX	29.115	-95.463	7.7	2006	2022

Table S6. Seasonal Theil-Sen slope analysis of selected ASOS and EPA stations across the HMA. Sen slopes are reported in per year in this table. Values containing an asterisk (*) indicate significance at $p < 0.05$.

ICAO:	KIAH	KGLS	KHOU	KEFD	KLVJ	KSGR	KCXO	KDWH	KAXH	KLBX
Max. Temp.	0.043*	0.049*	0.039*	-0.033*	0.038*	0.043*	0.044	0.047	-0.068	0.040*
Min. Temp.	0.060	0.066	0.062*	-0.07	0.064*	0.058*	0.054*	0.057*	-0.157*	0.062*

EPA:	482011039	482010058	482011035
Avg. PM2.5	-0.191*	-0.120*	-0.293*

Table S7. OLS Linear Regression equations for each season for Tmax across the HMA. Trained on 20% of the observed surface data from ASOS sites across the region. The final variable in each equation is the season coefficient.

Season	Tmax Regression
**DJF**	-100.840 + 0.437(LST Day)
**MAM**	-100.840 + 0.437(LST Day) – 0.113
**JJA**	-100.840 + 0.437(LST Day) + 3.110
**SON**	-100.840 + 0.437(LST Day) + 1.975

Table S8. Mean values of selected MODIS variables as well as surface Tmax and PM2.5 across the HMA urban census tracts, isolated by SVI bins.

MEAN	2000-2009 (2010-2019)						2010s-2000s Difference					
SVI Bin	LST Day	LST Night	NDVI	AOD	T_{max}	$PM_{2.5}$	LST Day	LST Night	NDVI	AOD	T_{max}	$PM_{2.5}$
**0.0-0.25**	300.48 (300.97)	289.83 (289.94)	0.49 (0.49)	0.15 (0.14)	304.71 (304.99)	11.39 (9.02)	1.023	0.456	-0.021	-0.011	0.407	-2.323
**0.25-0.50**	300.12 (300.78)	289.40 (289.80)	0.52 (0.50)	0.14 (0.14)	304.37 (304.66)	11.03 (8.72)	0.816	0.404	-0.018	-0.005	0.341	-2.306
**0.50-0.75**	300.39 (300.92)	289.73 (289.84)	0.49 (0.49)	0.15 (0.14)	304.61 (304.74)	11.25 (8.73)	0.750	0.370	-0.016	-0.010	0.318	-2.298
**0.75-1.0**	300.84 (301.83)	289.84 (290.28)	0.46 (0.45)	0.15 (0.15)	304.83 (305.41)	11.30 (9.36)	0.837	0.344	-0.019	-0.002	0.327	-2.236

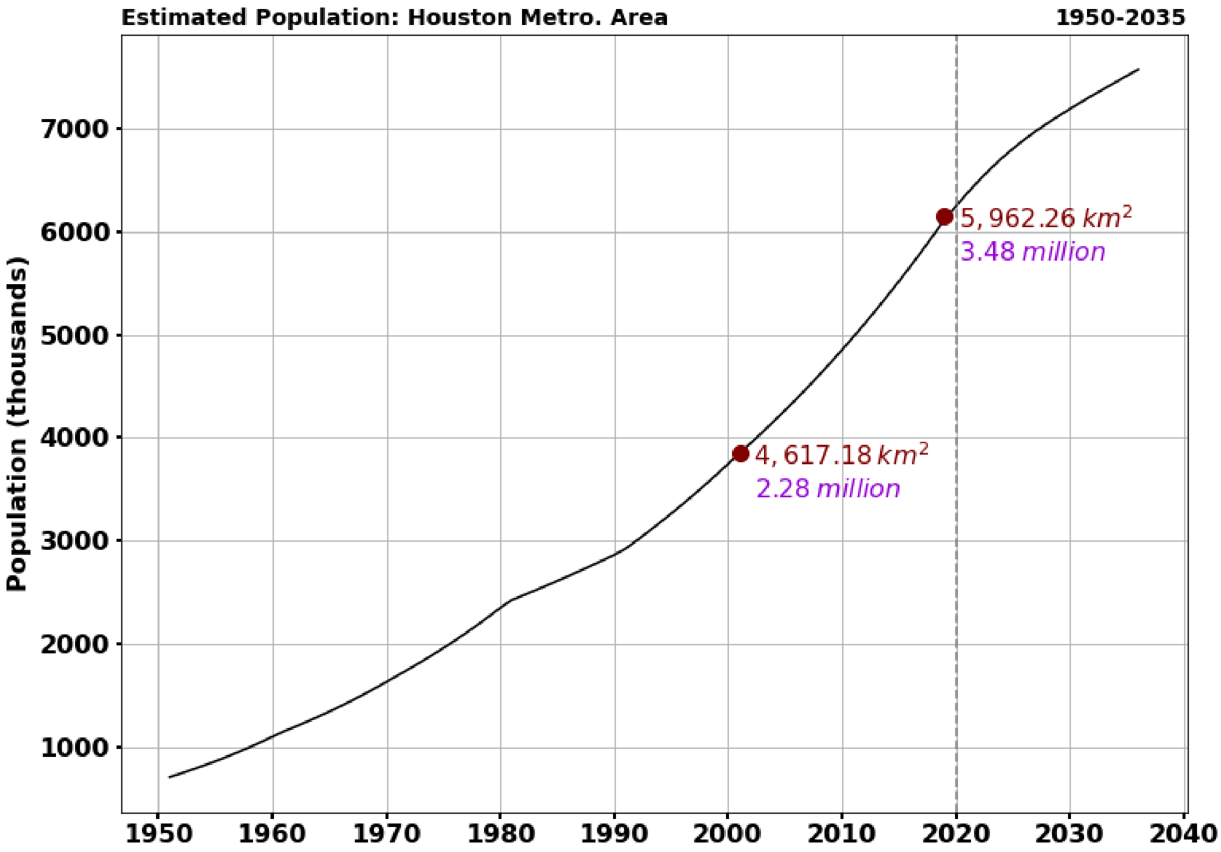

Figure S1. Census estimates and projected population growth (in thousands) for the Houston metropolitan area, 1955-2035 (US Census Bureau 2022; United Nations 2022). Red dots denote years 2001 and 2019 respectively and the area of urban land cover with HMA region for these specific years are shown next to the markers, where purple values are estimated population counts for Census block groups that are majority urban classified. Note that the area of urban land cover is computed using the NLCD dataset.

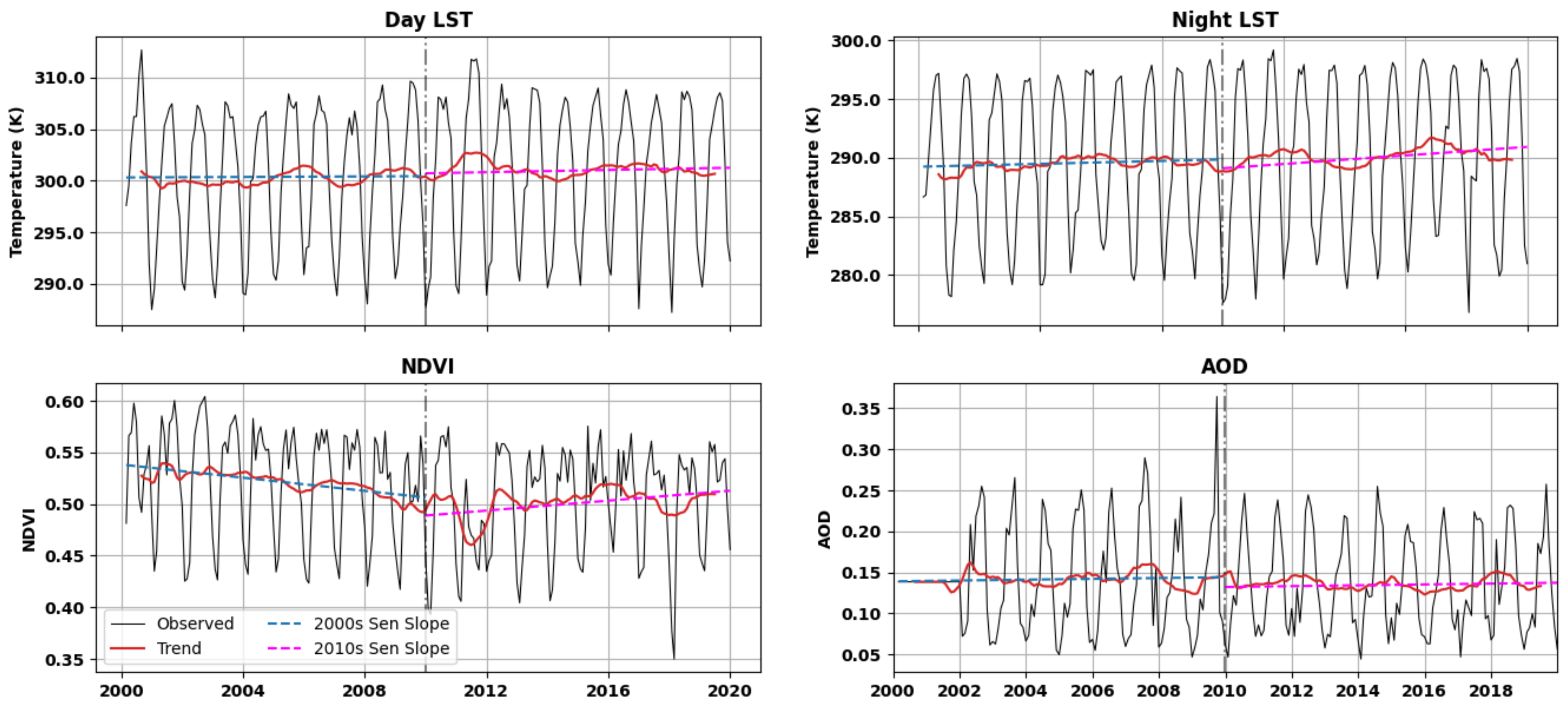

Figure S2. Monthly mean time series of selected MODIS variables, spatially averaged across the HMA, isolated to urban census tracts. The red lines are seasonally decomposed trends, and the blue and pink dashed lines are the Theil-Sen slopes for each of the decades 2000-2009 and 2010-2019.

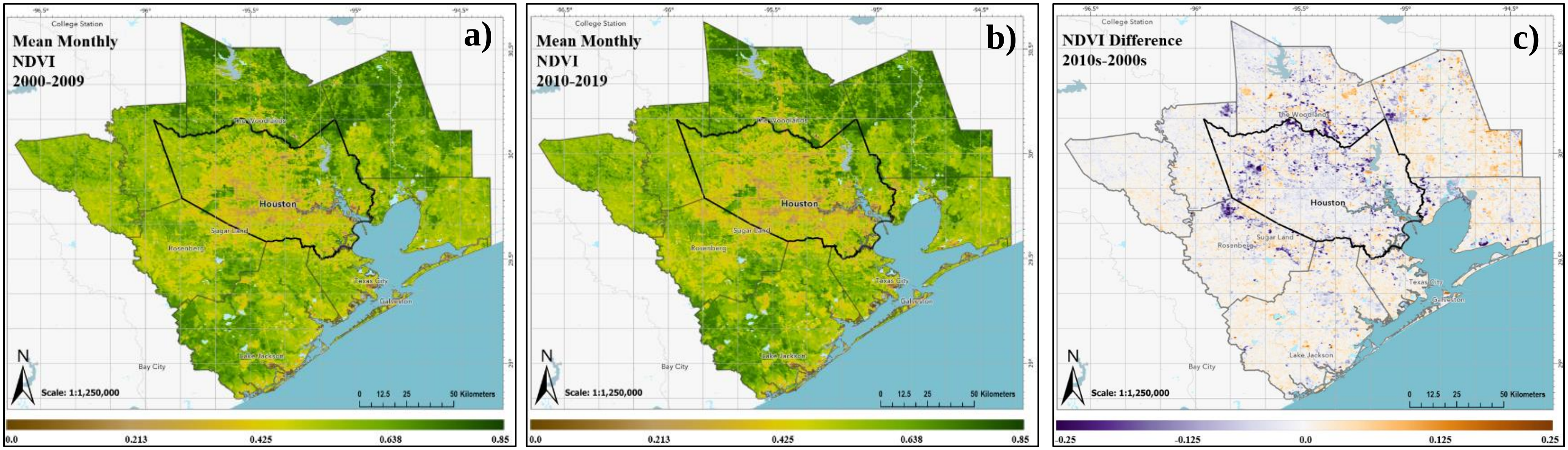

Figure S3. Decadal average MODIS-derived NDVI over the HMA for the periods a) 2000-2009, b) 2010-2019, and c) 2010-2019 minus 2000-2009 difference.

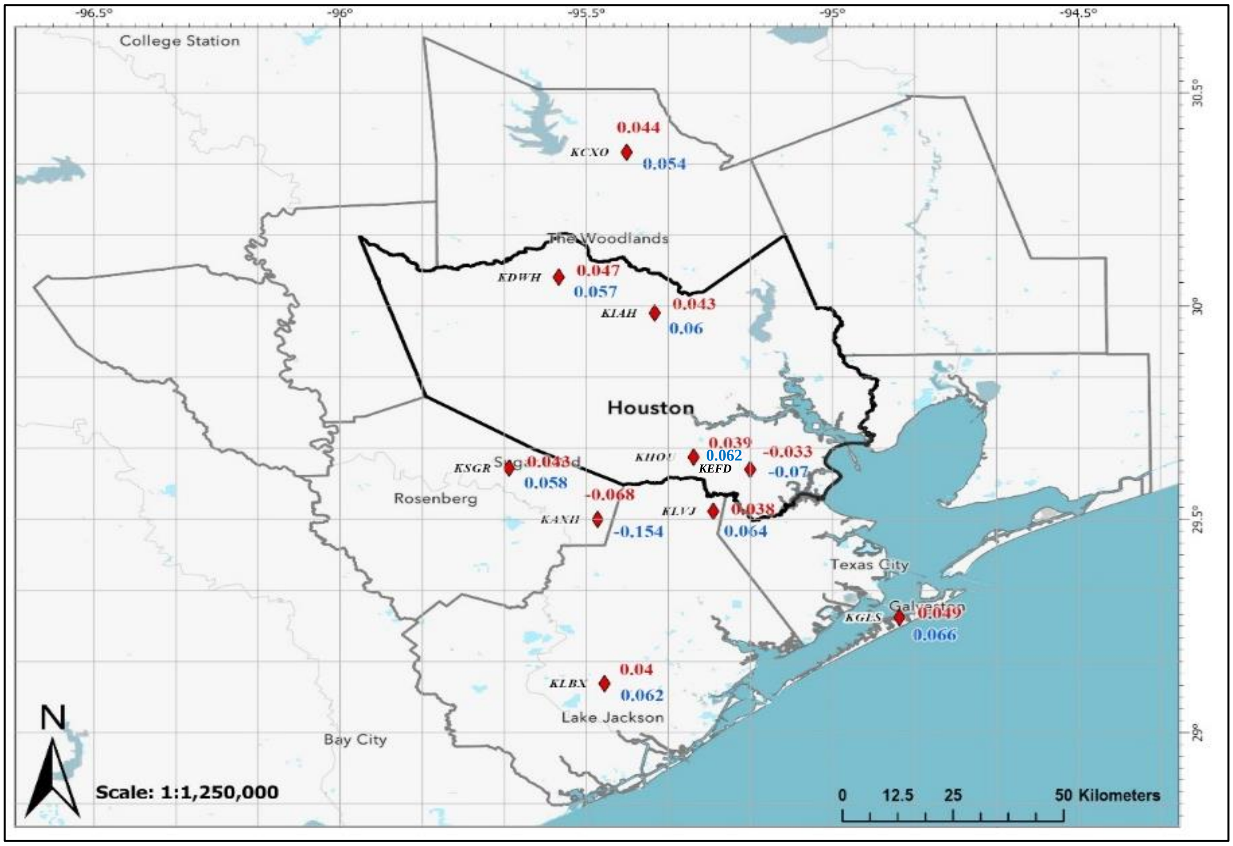

Figure S4. Spatial pattern of the seasonal Sen slopes computed per year for selected ASOS station Tmax (red) and Tmin (blue), with the numbers offset to the right of their respective ASOS location.

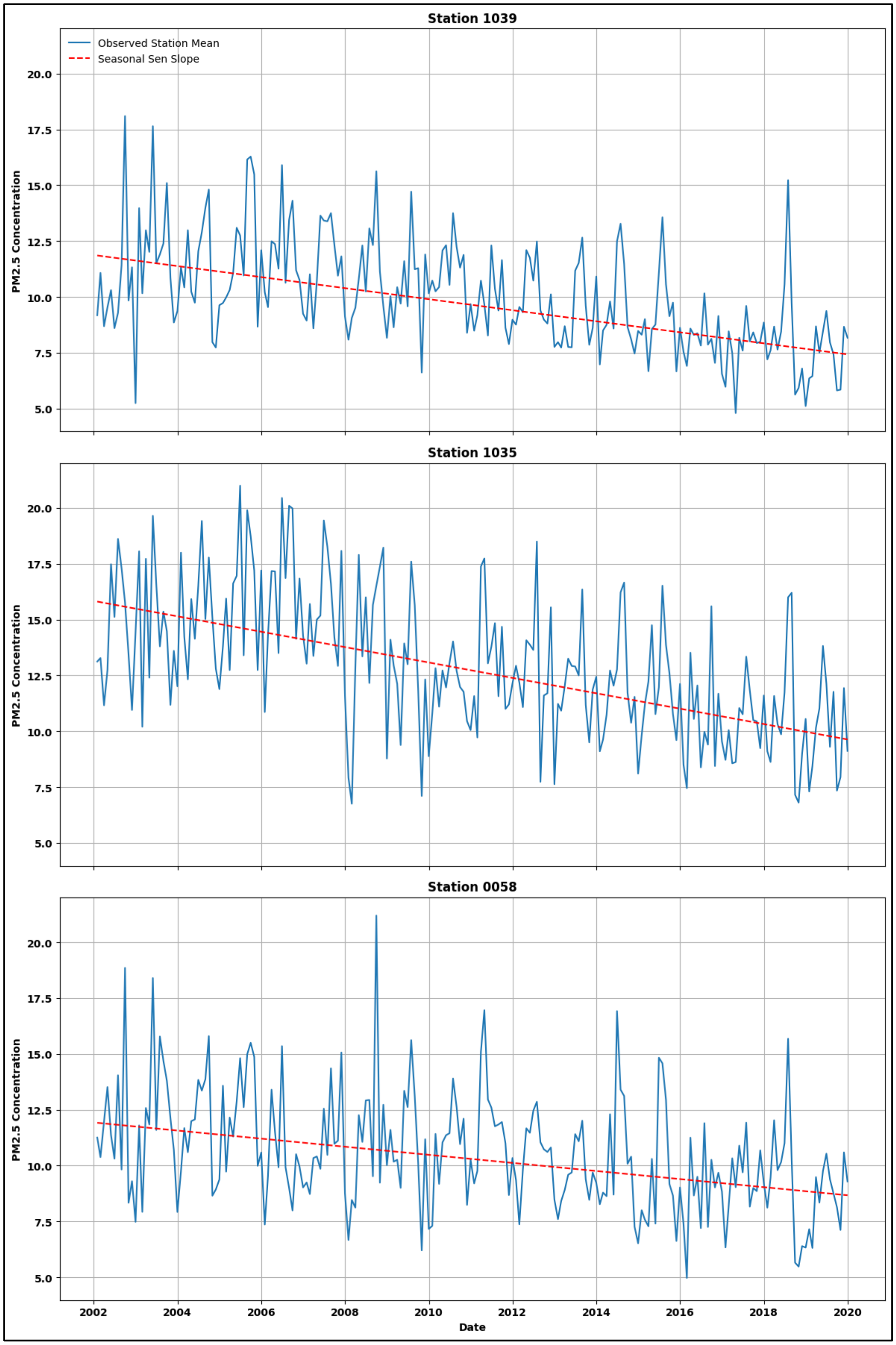

Figure S5. Monthly mean surface PN2.5 observations for three selected EPA sites across the HMA, 2002-2019.

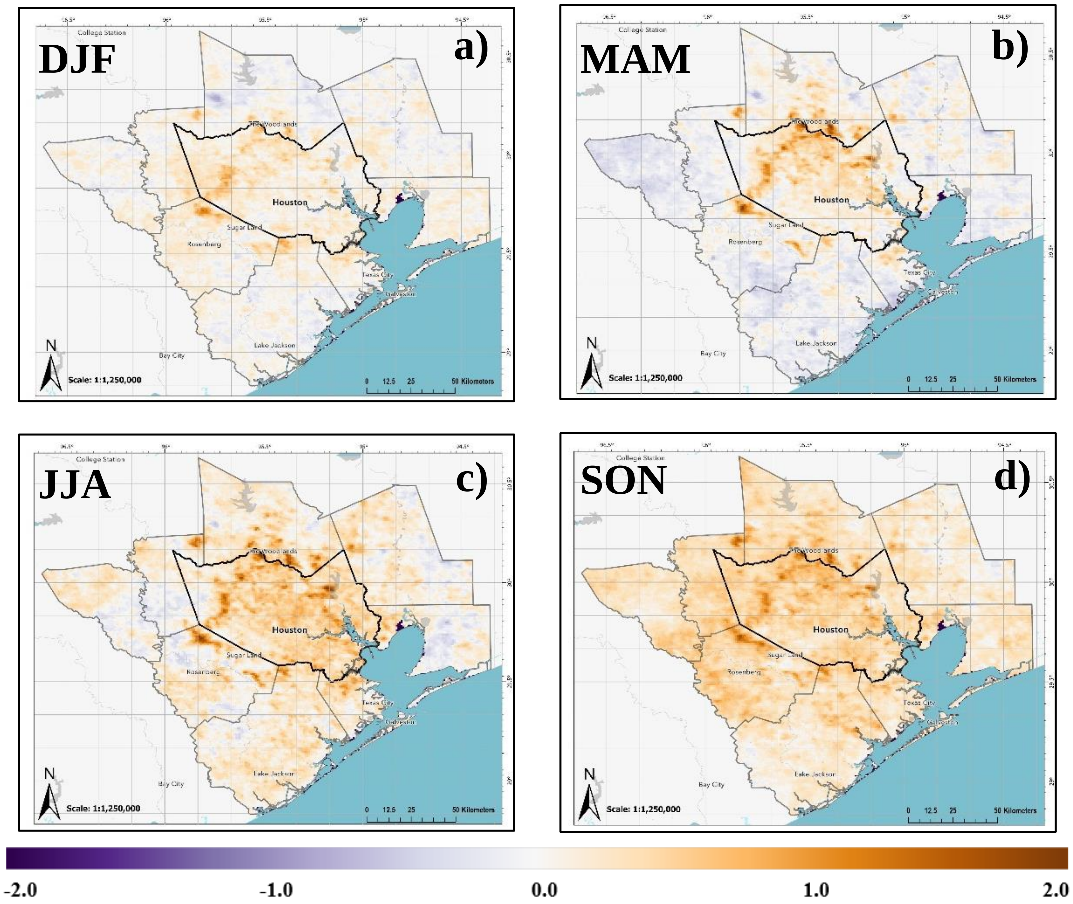

Figure S6. Predicted decadal difference (2010s-2000s) of $\mathbf{T_{max}}$ for each of the four seasons of a) DJF, b) MAM, c) JJA, and d) SON. Differences are reported in Kelvin.

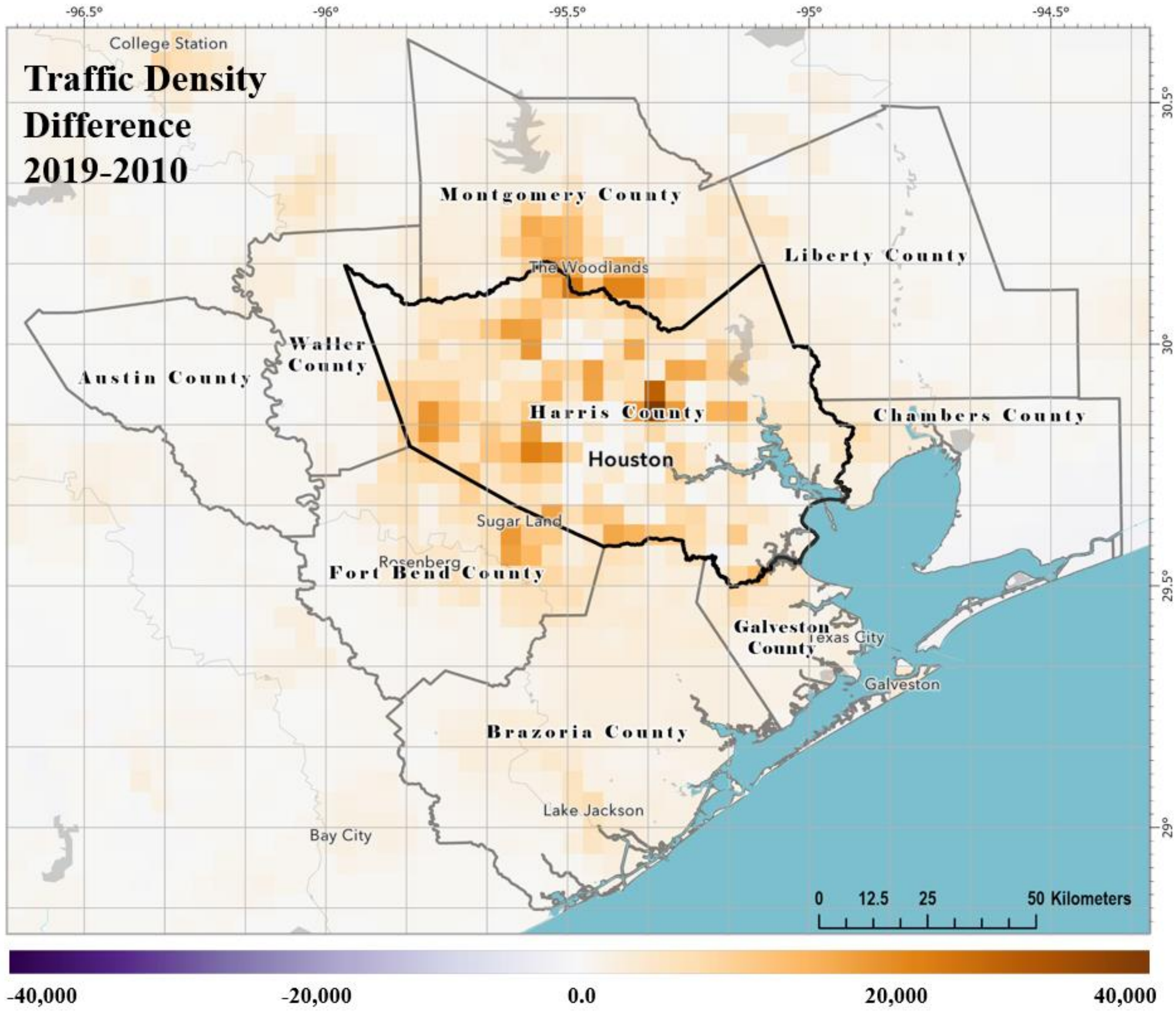

Figure S7. Spatially differenced annual traffic density (vehicles per day) reported by the Texas Department of Transportation, 2019 subtracted by 2010. The entire HMA shows an evident increase in traffic density, with highest concentrations in the newly urbanized regions (see Figure 3c).

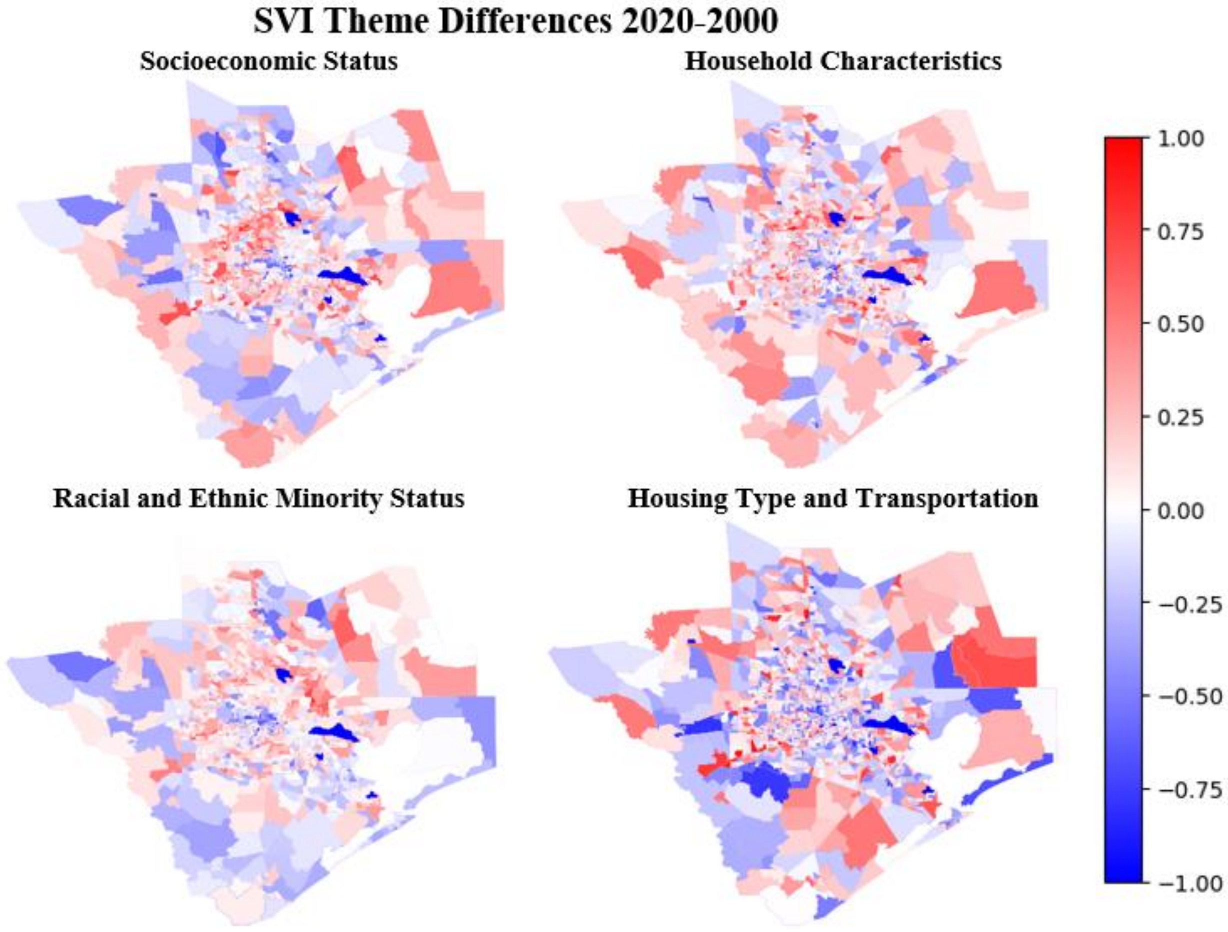

Figure S8. Spatial difference plots of each of the four main themes used to compute total SVI. These four main themes use a combined 16 census variables, and each of the four themes produce a value from 0-1 that is to be interpreted the same as the overall vulnerability (see section 3.3 of the main manuscript).
